# The impact of male age on embryo quality: a retrospective study using time-lapse imaging

**DOI:** 10.5935/1518-0557.20160041

**Published:** 2016

**Authors:** Guilherme R. F. Rosário, Diana S. Vidal, Adriana V. Silva, Antônio C. C. Franco

**Affiliations:** 1 Embryolife Reproductive Medicine Institute, São José dos Campos/SP

**Keywords:** Male age, time-lapse, morphokinetics, embryo quality

## Abstract

**Objective:**

This study aimed to correlate male age with embryo morphokinetic parameters on D3 considering the timing and the exact moment of embryo cleavage.

**Methods:**

Time-lapse imaging was used to produce an ideal cleavage curve for the embryos analyzed. The percentage of embryos under the curve was analyzed and correlated with male age.

**Results:**

32.6% of the embryos from patients aged 28-33 years were under the curve; 36.2% of the embryos from patients aged 34-39 years were under the curve; 41.3% of the embryos from patients aged 40-45 years were under the curve; and 26.3% of the embryos fro patients aged 46-57 years were under the curve.

**Conclusions:**

a statistically non-significant decrease was observed in the percentage of embryos under the optimal cleavage curve on D3 in the group of men aged between 40 and 45 years. Further studies looking into embryos in the blastocyst stage (D5 or D6) are required.

## INTRODUCTION

Male age has been associated with decreased semen quality (Schwartz *et al.*, 1983; Bujan *et al*., 1988; Silva *et al*., 2012; Oliveira *et al*., 2014.). It is also known that advanced paternal age is linked to higher rates of miscarriage (de la Rochebrochard & Thonneau., 2002; Slama *et al*., 2005; Kleinhaus *et al*., 2006), autosomal dominant diseases, aneuploidy, and other diseases (Glaser *et al*., 2003; Schmid *et al*., 2007). Other authors have associated advanced male age with greater sperm DNA fragmentation (Simon *et al*., 2014).

Abnormal sperm morphology and changes in embryo morphology have been closely linked, suggesting that sperm quality affects embryo development (Vagnini *et al*., 2007; Meng *et al.*, 2016.).

Time-lapse imaging has been used to monitor embryo development and help pick the best embryo based on cleavage kinetics (Meseguer *et al*., 2011; Cruz *et al*., 2012.; Herrero *et al*., 2013; Kirkegaard *et al*., 2012; 2013; Aguilar *et al*., 2014; Basile *et al.*, 2015). This imaging technique accurately predicts blastocyst formation (Motato *et al*., 2016), allowing early selection of embryos with high implantation potential within shorter periods of incubation (Milewski *et al*., 2015). Interestingly, prolonged embryo culture has been associated with significant epigenetic changes (Lonergan *et al*., 2003; Calle *et al*., 2012) and increased risk of preterm delivery when compared to embryos transferred on D2 or D3 (Maheshwari *et al*., 2013; Giving *et al*., 2014).

Considering the timing and exact moment of embryo cleavage described by Meseguer *et al.*, 2011, this study aimed to find whether male age correlated with embryo morphokinetic parameters on D3.

## MATERIALS AND METHODS

Two hundred and ninety-six embryos obtained from intracytoplasmic sperm injection (ICSI) procedures were included in the study.

The embryos were analyzed using time-lapse imaging (10/10 min), and the exact time of occurrence of significant embryo development events was noted.

### ICSI

ICSI was performed on culture medium containing HEPES. A Nikon^®^ Eclipse TE 2000-S microscope at 250x magnification was used. Temperature was controlled in the central vinyl surface of the micro-handler table with a Greisinger^®^ GMH 3230 surface thermometer (Germany) with validated calibration. After ICSI, the embryos were rinsed with the same culture medium in which they developed. Rinsing was carried out with at least three drops (~ 50mL) of pre-equilibrated medium. Then the oocytes were placed in micro-wells from the special time-lapse board and taken to an incubator.

### Incubation

The same culture medium was used for all embryos included in this study (Basile *-*., 2013). The CO2 level was as indicated by the manufacturer of the medium, while O2 levels were kept at ~ 20%.

Culture plates with nine or sixteen wells were prepared and pre-equilibrated in the incubator. After pre-equilibration, all micro-bubbles were carefully removed.

### Image acquisition system

The images were captured with a microscope camera placed inside a "big box" incubator type. Photos were taken every 10 minutes for the composition of a time line. The system used a green homogeneous LED light source.

### Morphokinetic parameter assessment based on time-lapse imaging

A software program was used to retrospectively analyze the images depicting the events that occurred after ICSI, and identify the precise moments at which pronuclei and cell walls disappeared and abnormalities arose.

An ideal cleavage curve plotted with the aid of analysis software was considered (Meseguer *et al*., 2011).

Graphic 1 illustrates embryo cleavage. A slight delay was observed in cleavage from three to four cells, which was enough to distinguish the embryos falling outside the optimal development curve.

**Graph 1:**
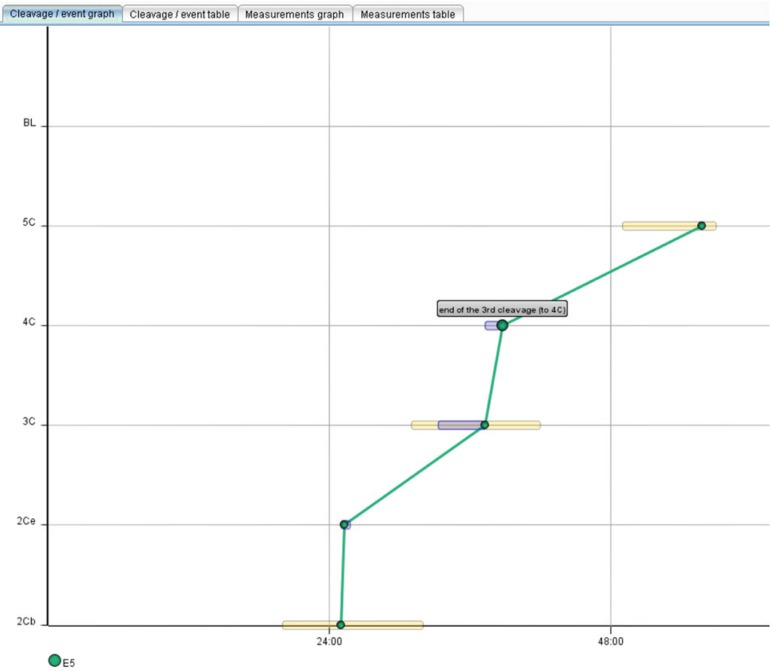
Embryo development curve.

### Female factor infertility

In order to mitigate the impact of female factor infertility, only the data from oocytes not presenting morphological abnormalities were analyzed (REDLARA 2006).

### Statistical analysis

Quantitative variables were described by means of measures of central tendency, scatter, and position, whereas male age was categorically described in terms of absolute and relative frequencies.

Binomial logistic regression with robust variance was used to check the impact of male age on the number of embryos (Mccullagh & Nelder, 1989). The software used in the analysis was the R (version 3.2.2).

## RESULTS

Table 1 shows the description of the studied variables.

**Table 1:** Description of the study variables.

Variables	Mean	SD	Min.	Q1	Q2	Q3	Max.
Male age	37.88	6.87	28.00	33.00	36.50	43.00	54.00
Embryos under the curve	1.79	1.93	0.00	0.00	1.00	3.00	9.00
Total Embryos	5.10	2.99	1.00	3.00	4.00	7.00	15.00
Percentage of embryos under the curve	35.1%	27.9%	3.00	3.00	33.3%	58.3%	100.0%

- Mean age was 37.88 years, with a standard deviation of 6.87 years.
- A mean of 1.79 embryos were under the curve; the minimum and maximum values were 0 and 9, respectively.
- The number of embryos ranged from 1 to 15; the mean number of embryos was 5.10.
- A mean of 35.1% of the embryos were under the curve.
- Table 2 shows the percentage of embryos under the age curve; in it, 41.3% of the embryos of patients aged between 40 and 45 years were under the curve, versus 26.3% of the embryos of patients aged between 46 and 57 years.
  **Table 2:** Percentage of Embryos Under the Curve by Age.

Age	Total Of Embryos	Embryos under the curve	% of embryos under the curve
28-33	89	29	32.6%
34-39	94	34	36.2%
40-45	75	31	41.3%
46-57	38	10	26.3%
- Table 3 shows the binomial logistic regression with robust variance (Mccullagh & Nelder, 1989) adjusted to check for the impact of male age on the number of embryos under the curve. The following conclusions may be derived:
  **Table 3:** Impact of Male Age on the Number of Embryos Under the Curve

Source	β	E.P.(β)	p-value	OR	95% CI
intercept	-0.727	0.294	0.016	-	-
Male age = 28-33				1	-
Male age = 34-39	0.159	0.405	0.696	1.17	[0.53;2.59]
Male age = 40-45	0.377	0.423	0.377	1.46	[0.64;3.34]
Male age = 46-57	-0.303	0.561	0.592	0.74	[0.25;2.2]
- The chance of an individual aged 34-39 having an embryo under of the curve was 1.17 [0.53; 2.59] times the chance of an individual aged 28-33 years, but this difference was not statistically significant (*P*-value = 0.696).
- The chance of an individual aged 40-45 having an embryo under of the curve was 1.46 [0.64; 3.34] times the chance of an individual aged 28-33 years, but this difference was not statistically significant (*P*-value = 0.377).
- The chance of an individual aged 46-57 having an embryo inside of the curve was 0.76 [0.25; 2.22] times the chance of an individual aged 28-33 years, but this difference was not statistically significant (*P*-value = 0.592).
- A comparison against the findings on Table 3 shows the following:
- The chance of an individual aged 40-45 having an embryo under of the curve was 1.24 [0.54; 2.84] times the chance of an individual aged 34-39 years, but this difference was not statistically significant (*P*-value = 0.600).
- The chance of an individual aged 46-57 having an embryo inside of the curve was 0.63 [0.21; 1.91] times the chance of an individual aged 34-39 years, but this difference was not statistically significant (*P*-value = 0.408).
- The chance of an individual aged 46-57 having an embryo inside of the curve was 0.51 [0.16; 1.58] times the chance of an individual aged 40-45 years, but this difference was not statistically significant (*P*-value = 0.236).

**Graph 2:**
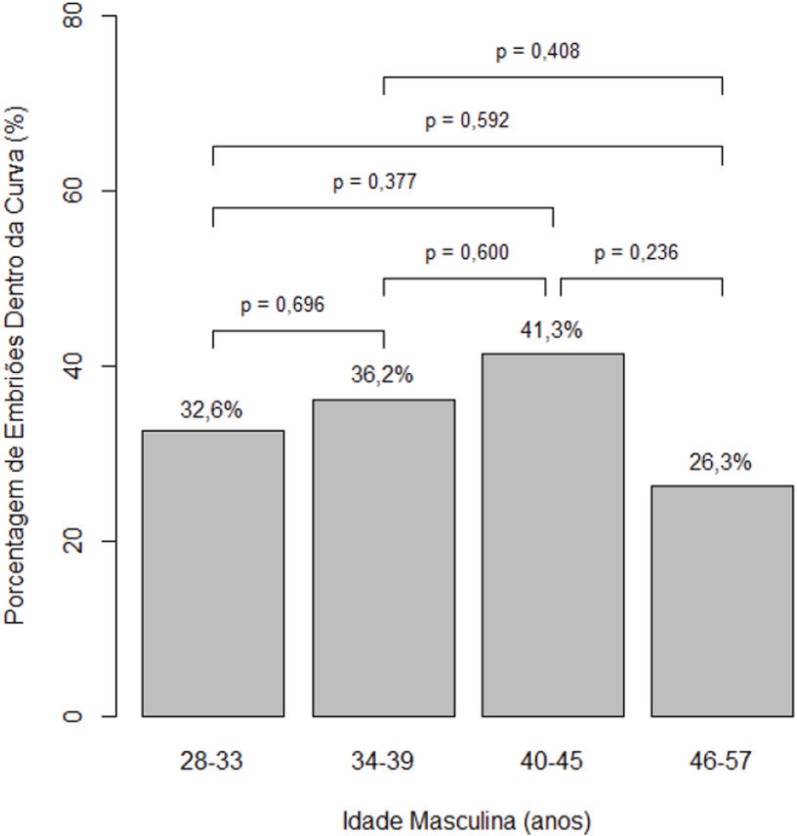
Percentage of embryos under the curve for each age group

Graph 2 shows the percentage of embryos under the curve for each age group with *P*-values estimated by binomial logistic regression with robust variance, as shown in Table 3.

## DISCUSSION

Various different aspects concerned with the impact of male age on semen quality have been described in the literature. Some studies have shown an inverse correlation between male age and semen volume - volume decreasing with age - (Spandorfer *et al.*, 1998; Andolz *et al*., 1999; Moskovtsev *et al*., 2009; Brahem *et al.*, 2011; Oliveira *et al.*, 2014), sperm motility (Moskovtsev *et al.*, 2009; Brahem *et al.*, 2011; Dain *et al.*, 2011; Stone *et al*., 2013; Oliveira *et al*., 2014) and sperm vitality (Moskovtsev *et al*., 2009; Brahem *et al*., 2011; Zhu *et al*., 2011; Stone *et al*., 2013). Conversely, other authors failed to observe connections between any such semen parameters and paternal age (Berling *et al*., 1997; Spandorfer *et al.*, 1998; Frattarelli *et al*., 2008; Nijs *et al.*, 2011; Fréour *et al*., 2012). Some studies found no correlation between male age and semen concentration (Spandorfer *et al.*, 1998; Frattarelli *et al*., 2008; Bellver *et al*., 2008; Dain *et al*., 2011; Nijs *et al.,* 2011; Fréour *et al.*, 2012), whereas other authors have either described decreases (Luna *et al*., 2009; Stone *et al*., 2013) or increases (Andol *et al*., 1999; Brahem *et al*., 2011) in semen concentration over time. The studies cited above generally differ over their conclusions and some do not specify a number of points such as whether the group of enrolled patients includes solely individuals seen at ART clinics, how many of them smoke or drink alcohol, have varicocele, or are taking medication or vitamins. These differences complicate the interpretation of results.

Discrepant findings have been reported in the literature in regards to sperm nuclear vacuoles. Some studies have identified significant correlations between male age and sperm nuclear vacuoles (Braga *et al.*, 2011; Silva *et al.*, 2012; Oliveira *et al.*, 2014). However, two of the authors (Braga *et al.*, 2011; Silva *et al.*, 2012) reported that there was no correlation between normal sperm frequency and male age defined by MSOME (motile sperm organelle morphology examination).

Authors correlating paternal age and embryo development have claimed that embryo morphology during cleavage is not affected by male age (Frattarelli *et al*., 2008) and that male age is irrelevant for the outcome ART procedures (Bellver *et al*., 2008), while others believe there is not enough data to support such claim (Dain *et al*., 2011). However, a significant decrease in blastocyst formation was observed with increasing age (Luna *et al*., 2009; Dain *et al*., 2011), probably reflecting the paternal genome activation in the embryo.

All previous articles analyzed male age in relation to embryo quality by considering exclusively embryo morphology criteria. It is important to remember that morphologically identical embryos may be assessed or fall into the exclusion criteria according to the algorithm proposed by Basile *et al.* (2015). Events related to low implantation rates such as multinucleation (Pickering *et al*., 1995), asymmetric blastomeres (Hardarson *et al.,* 2001), direct cleavage to three cells (1C-3C) (Rubio *et al*., 2012) and asynchronous disappearance of pronuclei (Rosário *et al.,* 2015) may be difficult or impossible to observe without the aid of time-lapse imaging.

Table 2 and Graph 2 in this study describe decreased percentages of embryos under the normal cleavage curve, as also shown by other authors. This finding was noted in patients approaching the fifth decade of life. However, statistical tests showed that such decrease was not significant. This finding was also reported in other studies (Gallardo *et al.*, 1996; Aboulghar *et al*., 2007; Frattarelli *et al*., 2008; Bellver *et al.*, 2008; Dain *et al.*, 2011).

Semen quality may decrease with advanced age, but the actual impact of male age on embryo viability is multifactorial.

## CONCLUSION

Decreased percentages of embryos under the normal cleavage curve on D3 were found for males aged 45 years and older, but such difference was not statistically significant. Further studies are required to assess the status of embryos on the blastocyst stage (D5 or D6).
